# A classification prognostic score to predict OS in stage IV well-differentiated neuroendocrine tumors

**DOI:** 10.1530/ERC-17-0489

**Published:** 2018-03-20

**Authors:** Sara Pusceddu, Francesco Barretta, Annalisa Trama, Laura Botta, Massimo Milione, Roberto Buzzoni, Filippo De Braud, Vincenzo Mazzaferro, Ugo Pastorino, Ettore Seregni, Luigi Mariani, Gemma Gatta, Maria Di Bartolomeo, Daniela Femia, Natalie Prinzi, Jorgelina Coppa, Francesco Panzuto, Lorenzo Antonuzzo, Emilio Bajetta, Maria Pia Brizzi, Davide Campana, Laura Catena, Harry Comber, Fiona Dwane, Nicola Fazio, Antongiulio Faggiano, Dario Giuffrida, Kris Henau, Toni Ibrahim, Riccardo Marconcini, Sara Massironi, Maja Primic Žakelj, Francesca Spada, Salvatore Tafuto, Elizabeth Van Eycken, Jan Maaten Van der Zwan, Tina Žagar, Luca Giacomelli, Rosalba Miceli, NEPscore Working Group, NEPscore Working Group, Francesca Aroldi, Alberto Bongiovanni, Rossana Berardi, Nicole Brighi, Sara Cingarlini, Carolina Cauchi, Federica Cavalcoli, Carlo Carnaghi, Francesca Corti, Marilina Duro, Maria Vittoria Davì, Chiara De Divitiis, Paola Ermacora, Anna La Salvia, Gabriele Luppi, Giuseppe Lo Russo, Federico Nichetti, Alessandra Raimondi, Vittorio Perfetti, Paola Razzore, Maria Rinzivillo, Sabine Siesling, Martina Torchio, Boukje Van Dijk, Otto Visser, Claudio Vernieri

**Affiliations:** 1 Department of Medical Oncology ENETS Center of ExcellenceFondazione IRCCS Istituto Nazionale dei Tumori di Milano, Milan, Italy; 2 Unit of Clinical Epidemiology and Trial OrganizationFondazione IRCCS Istituto Nazionale dei Tumori di Milano, ENETS Center of Excellence, Milan, Italy; 3 Department of Preventive and Predictive MedicineFondazione IRCCS Istituto Nazionale dei Tumori di Milano, Evaluative Epidemiology Unit, ENETS Center of Excellence, Milan, Italy; 4 Department of PathologyFondazione IRCCS Istituto Nazionale dei Tumori, Milano, ENETS Center of Excellence, Milan, Italy; 5 University of MilanMilan, Italy; 6 Liver SurgeryTransplantation and Gastroenterology, University of Milan and Istituto Nazionale Tumori Fondazione IRCCS, ENETS Center of Excellence, Milano, Milan, Italy; 7 Department of Thoracic Surgical OncologyFondazione IRCCS Istituto Nazionale dei Tumori, Milano, ENETS Center of Excellence, Milan, Italy; 8 Department of Nuclear Medicine ENETS Center of ExcellenceFondazione IRCCS Istituto Nazionale dei Tumori, Milano, Milan, Italy; 9 Department of Medical GastroenterologyAzienda Ospedaliera Sant’Andrea, Roma ENETS Center of Excellence, Rome, Italy; 10 Department of Medical OncologyAzienda Ospedaliera Universitaria Careggi, Firenze, Italy; 11 Department of Medical OncologyPoliclinico di Monza, Monza, Italy; 12 Department of Medical OncologyAzienda Ospedaliera Universitaria San Luigi Gonzaga, Orbassano, Italy; 13 Department of Medical OncologyPoliclinico Sant’Orsola Malpighi, Bologna, Italy; 14 Ireland National Cancer RegistryCork, Ireland; 15 Department of Medical OncologyIEO – Istituto Europeo di Oncologia, Milano, ENETS Center of Excellence, Milan, Italy; 16 Department of Thyroid and Parathyroid Surgery UnitAzienda Ospedaliera Universitaria Federico II, ENETS Center of Excellence, Naples, Italy; 17 Department of Medical OncologyIOM – Istituto Oncologico del Mediterraneo, Catania, Italy; 18 Belgian Cancer RegistryBrussels, Belgium; 19 Osteoncology and Rare Tumors CenterIstituto Scientifico Romagnolo per lo Studio e la Cura dei Tumori IRST, IRCCS, Meldola, Italy; 20 Department of Medical OncologyOspedale Santa Chiara, Pisa, Italy; 21 Gastroenterology and Endoscopy UnitFondazione IRCCS Ospedale Maggiore Policlinico, Milan, Italy; 22 Institute of Oncology LjubljanaEpidemiology and Cancer Registry, Ljubljana, Slovenia; 23 Department of Medical OncologyFondazione IRCCS Pascale, ENETS Center of Excellence, Naples, Italy; 24 Department of ResearchNetherlands Comprehensive Cancer Organisation (IKNL), Utrecht, The Netherlands; 25 Department of Surgical Sciences and Integrated DiagnosticsUniversity of Genoa, Genoa, Italy

**Keywords:** neuroendocrine tumors, overall survival, prognosis, prognostic score, validation

## Abstract

No validated prognostic tool is available for predicting overall survival (OS) of patients with well-differentiated neuroendocrine tumors (WDNETs). This study, conducted in three independent cohorts of patients from five different European countries, aimed to develop and validate a classification prognostic score for OS in patients with stage IV WDNETs. We retrospectively collected data on 1387 patients: (i) patients treated at the Istituto Nazionale Tumori (Milan, Italy; *n* = 515); (ii) European cohort of rare NET patients included in the European RARECAREnet database (*n* = 457); (iii) Italian multicentric cohort of pancreatic NET (pNETs) patients treated at 24 Italian institutions (*n* = 415). The score was developed using data from patients included in cohort (i) (training set); external validation was performed by applying the score to the data of the two independent cohorts (ii) and (iii) evaluating both calibration and discriminative ability (Harrell C statistic). We used data on age, primary tumor site, metastasis (synchronous vs metachronous), Ki-67, functional status and primary surgery to build the score, which was developed for classifying patients into three groups with differential 10-year OS: (I) favorable risk group: 10-year OS ≥70%; (II) intermediate risk group: 30% ≤ 10-year OS < 70%; (III) poor risk group: 10-year OS <30%. The Harrell C statistic was 0.661 in the training set, and 0.626 and 0.601 in the RARECAREnet and Italian multicentric validation sets, respectively. In conclusion, based on the analysis of three ‘field-practice’ cohorts collected in different settings, we defined and validated a prognostic score to classify patients into three groups with different long-term prognoses.

## Introduction

Neuroendocrine tumors (NETs) are a heterogeneous class of neoplasms with increasing incidence worldwide (Fraenkel *et al*. 2012, Dasari *et al*. 2017). A number of factors including tumor histology, primary site, staging and proliferative index influence tumor behavior and patients’ survival (Panzuto *et al*. 2014).

Patients with NETs are classified according to both tumor morphology and assessment of proliferation according to World Health Organization (WHO)/European Neuroendocrine Tumor Society (ENETS) guidelines. Morphology is classified as well- or poorly-differentiated; grading (G1–G2–G3) is assessed by Ki-67 and mitotic count (Rindi *et al*. 2010, Bosman 2010). In particular, well-differentiated (WD) NETs are considered indolent malignancies and are associated with a relatively favorable prognosis (Öberg *et al*. 2012, van der Zwan *et al*. 2013, Frilling *et al*. 2014). However, even WDNETs present a marked heterogeneity in their clinical behavior (Pusceddu *et al*. 2017). Given this variable course of disease, selection of the most suitable treatment (first-line and sequence) remains challenging.

Therefore, the development of prognostic scores able to classify patients according to clinical outcomes appears of the highest interest in current clinical research. Indeed, such scores may help guide treatment selection and, at the same time, could be used in the design of clinical trials (Mariani *et al*. 2005, Motzer *et al*. 2008). Prognostic scores for OS have been specifically developed to assess OS in patients with gut NETs (Modlin *et al*. 2010) or gastrointestinal high-grade, G3 neuroendocrine carcinomas (GI-NECs) (Lamarca *et al*. 2017), to predict progression-free survival of patients with stage IV NETs (Panzuto *et al*. 2017), or to predict disease recurrence rate after surgery in G1–G2 NETs (Genç *et al*. 2017). However, to our knowledge, no validated score addresses the prognosis of WDNETs in terms of OS.

In this large study, conducted in three independent cohorts of patients from five different European countries, we aimed to develop and validate a classification prognostic score for OS in patients with stage IV WD G1–G2 NETs.

## Patients and methods

### Study design

Three retrospective cohorts were included in this study: (i) a training cohort of patients treated at the Istituto Nazionale Tumori (INT) (Milan, Italy), a referral Center for the treatment of oncological disease and an European Neuroendocrine Tumor Society (ENETS) Center of Excellence for the treatment of gastroenteropancreatic (GEP)-NETs; (ii) a European external validation cohort, which comprised rare NET patients included in the database of the pilot study of the European project RARECAREnet (Gatta *et al*. 2017), which collected data on rare NETs of any site (poorly-differentiated NET of the lung were not in the database since they are not considered rare tumors) from 4 population-based cancer registries from 4 countries: Belgium, Slovenia, The Netherlands and Ireland and (iii) an Italian external validation multicentric cohort including only pancreatic NET (pNETs) patients treated at 24 different Italian institutions. Approval for data collection was obtained independently by each institution involved as per local practice.

### Description of the three cohorts

#### Training cohort

Out of a prospectively collected monocentric database including 1091 patients presenting with diagnosis of NET from 1988 to 2012 at INT, data of 515 patients were extracted to perform the present study if they presented G1–2 metastatic (stage IV) WD GEP-NETs or lung NETs at diagnosis. In more detail, the following primary sites were considered: (I) pancreatic neuroendocrine tumors, (II) midgut NETs (ileum appendix, caecum, jejunum, ileum, duodenum); (III) other GEP-NETs (stomach, rectum and colon except caecum); (IV) lung typical or atypical NETs and (V) NET of unknown primary.

NET diagnosis was confirmed at general hematoxylin and eosin staining histology and immunohistochemistry, in all cases by a dedicated pathologist (MM). Slices were reviewed for morphology, mitotic count and grading assessment in agreement with the 2010 GEP-NET WHO and 2015 lung NET WHO classifications (Rindi *et al*. 2010, Travis *et al*. 2015).

Patients were ineligible if they had a poorly-differentiated neuroendocrine carcinoma (NEC G3) or had other histology such as Merkel cell carcinomas, pheochromocytoma/paragangliomas, large cell NEC (LCNC) and small-cell lung cancer (SCLC).

We investigated the prognostic impact on survival of different clinical parameters, including age, gender, site of primary tumor, resection of primary tumor, metastatic site, time to metastasis development, functioning or not functioning status. All patients were followed up until the end of 2015.

#### External validation cohorts

The same inclusion criteria applied in the training cohort were used to identify patients for inclusion in the two external validation cohorts (European and Italian multicentric cohorts).

The European cohort was extracted from the RARECAREnet pilot study. All patients were stage IV at diagnosis with G1–2 grading score WD GEP-NETs, WD of the lung and unknown primary site cancers were selected. In total, we included 457 patients: 155 diagnosed in Belgium in the period 2004–2007; 168 in The Netherlands in the period 2005–2007 and 79 in Ireland and 55 in Slovenia in the period 2000–2007. All patients were followed up for vital status until the end of 2012. Therefore, patients from Netherlands had only seven years of follow-up, Belgium eight years, Ireland and Slovenia 10 years (only two patients contributing).

To externally validate the score in a selected cohort of patients with a single primary tumor site, we identified an Italian multicentric series of 415 patients with WD pNETs treated at 24 Italian Institutions from 2000 to 2015. All patients were followed up until the beginning of 2017.

### Statistical methods

The study endpoint was OS; the time was calculated from the date of diagnosis to the date of death from all causes, with censoring at the date of last follow-up in living patients. OS curves were estimated by the Kaplan–Meier method, with the log-rank test used to compare subgroups.

The NeuroEndocrine Prognostic Score classification (NEP-Score) for stage IV WD NET patients was developed using the data of patients included in the training set. Among the data made available in the institutional database, the *a priori* chosen putative prognostic covariates were patients’ age at metastasis detection (≤45, 46–65, >65 years), gender, site of primary tumor (ileum; lung; pancreas; other GEP-NETs – stomach, rectum and colon except caecum, unknown primary site), site of metastasis (single hepatic lesion; single extra-hepatic lesion; multiple hepatic lesions and multiple extra-hepatic lesions), time to metastasis development (synchronous, metachronous ≤24 months, metachronous >24 months), Ki-67 (MIB-1) (0–2, 3–20, not specified), functional status (yes vs no) and primary tumor resection (yes, no). Multivariable Cox model analysis was carried out and a covariate backward selection procedure based on the Akaike Information Criterion was applied (Akaike 1973). No interactions between covariates were assessed in the selection procedure. Such a procedure led to exclude gender and site of metastasis from the initial set of 8 parameters. NEP-Score was intended to classify patients according to their predicted 10-year OS. Thus, we firstly derived a covariate scoring system based on the 10-year OS predicted by the final Cox model. Then, a three-level prognostic score was derived for classifying patients according to their predicted 10-year OS: (i) favorable risk group: OS ≥70%; (ii) intermediate risk group: OS ≥30% and <70%; (iii) poor risk group: OS <30%.

NEP-Score performance was evaluated by examining calibration (calibration plot) and discriminative ability (Harrell C index) (Harrell *et al*. 1996) on the training set (internal validation). External validation was performed by applying the NEP-Score to the data of the two independent validation cohorts and evaluating both calibration and discriminative ability. To obtain the calibration plot in the testing cohorts and to be coherent with the score predictions, ideally, we should have used the 10-year OS probabilities predicted according to the final Cox model fitted in the training set. However, while in the Italian multicentric series, the percentage of patients still at risk at 10 years was as high as 17%, in the RARECAREnet cohort, it dropped to 3% at 10 years but was similar to that of the Italian series at 7 years (16%). Thus, in the latter cohort, we truncated the OS curves at 7 years, and we used the same cut-off for evaluating the NEP-Score performance.

Statistical analyses were conducted using SAS software (SAS Institute, Cary, NC, USA) and R software (http://www.r-project.org/).

Additional details are reported in the Supplementary Materials and methods (see section on supplementary data given at the end of this article).

## Results

**Figure 1:**
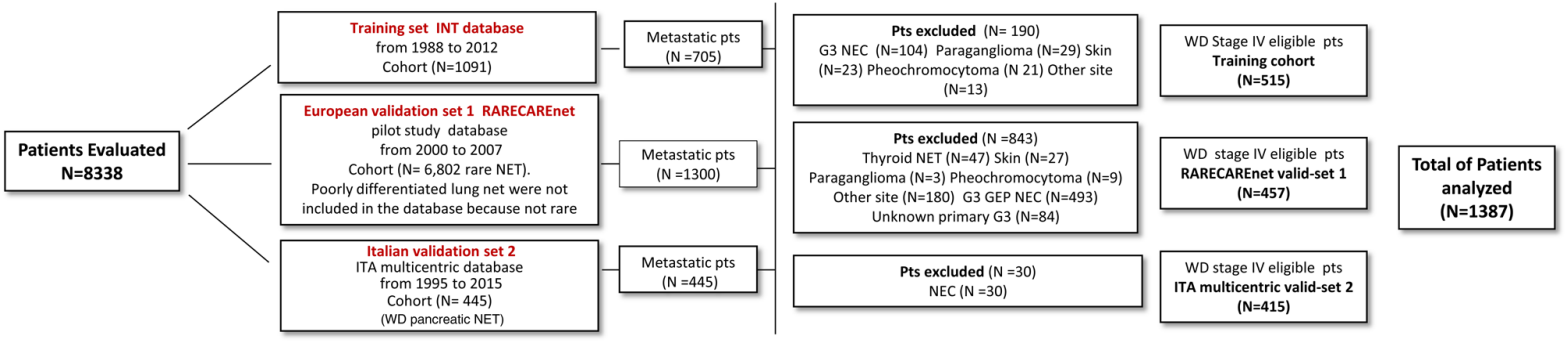
Patients’ disposition in the three cohorts. GEP, gastroenteropancreatic; INT, Istituto Nazionale Tumori; NEC, neuroendocrine carcinoma; NET, neuroendocrine tumors; Pts, patients; WD, well differentiated. A full colour version of this figure is available at 10.1530/ERC-17-0489.

Figure 1 displays the disposition of patients through the study period. Median follow-up (interquartile range, IQR) was 78 (36–131) months in the training set and 87 (75–102) and 77 (37–130) months in the European and Italian validation cohort, respectively. Table 1 depicts patient characteristics.

**Table 1:** Characteristics of 515 training set patients, 457 RARECAREnet cohort patients and 414 Italian series cohort patients.

	Training set No. (%)	RARECAREnet cohort No. (%)	Italian multicentric cohort No. (%)
Year of diagnosis			
<2000	159 (30.9)	–	18 (4.3)
≥2000	356 (69.1)	457 (100.0)	426 (95.7)
Gender			
Male	284 (55.1)	239 (52.3)	226 (54.5)
Female	231 (44.9)	218 (47.7)	189 (45.5)
Age at metastasis (years)			
Median, IQR	57, 45–65	65, 56–64	56, 45–65
<45	129 (25.0)	30 (6.6)	104 (25.1)
46–65	269 (52.2)	204 (44.6)	209 (50.4)
>65	117 (22.7)	223 (48.8)	102 (24.6)
Primary tumor classification site			
Other GEP-NET^+^	81 (15.7)	192 (42.0)	–
Lung (typical and atypical carcinoids)	69 (13.4)	54 (11.8)	–
Ileum	135 (26.2)	92 (20.1)	–
Pancreatic	139 (27.0)	58 (12.7)	415 (100.0)
Unknown	91 (17.7)	61 (13.3)	–
Functioning status			
Yes	123 (23.9)	14 (3.1)	71 (17.1)
No	392* (76.1)	443 (96.9)	344 (82.9)
Chromogranine A (pathological level ULN)			
Yes	56 (10.9)	–	225 (54.2)
No	185 (35.9)	–	143 (34.5)
Not performed	209 (40.6)	–	34 (8.2)
Unknown	65 (12.6)	–	13 (3.1)
Ki-67 (MIB-1)			
0–2	206 (40.0)	347 (75.9)	117 (28.2)
3–20	149 (28.9)	110 (24.1)	290 (69.9)
Missing	160 (31.1)	–	8 (1.9)
Primary tumor surgery			
Yes	298 (57.9)	234 (51.2)	236 (56.9)
No	217 (42.1)	223 (48.8)	179 (43.1)
Metastasis			
Syncronous	447 (86.8)	457 (100.0)	296 (71.3)
Metachronous ≤24 months	27 (5.2)	–	41 (9.9)
Metachronous >24 months	41 (8.0)	–	78 (18.8)
Metastasis site (stage IV)			
Liver (single metastasis)	18 (3.5)	–	158^†^ (38.1)
Liver (multiple metastasis)	212 (41.2)	–	–
Nodes (single site of metastases)	9 (1.7)	–	14^†^ (3.4)
Nodes	37 (7.2)	–	–
Lung^†^	12 (2.3)	–	–
Other (single site of metastases)	16 (3.1)	–	10 (2.4)
Multiple site (including liver)	189 (36.7)	–	220 (53.0)
Multiple site (excluding liver)	22 (4.3)	–	10 (3.1)
Peptide receptor radionuclide therapy			
Yes	37 (7.2)	–	104 (25.1)
No	478 (92.8)	–	311 (74.9)

*4 missing data imputed with modal value; ^+^stomach, rectum and colon except caecum; ^†^single or multiple.

IQR, interquartile range; ULN, upper level of normality.

In the training set, 218 patients died for any cause; 5- and 10-year OS (95% CI) was 64.3% (59.8–69.2%) and 42.6% (37.0–49.0%), respectively. In the European validation cohorts, 330 patients died for any cause; 5- and 7-year OS was 38.7% (34.5–43.5%) and 29.6% (25.5–34.3%). In the Italian validation set, 122 patients died for any cause; 5- and 10-year OS was 81.2% (76.9–85.6%) and 54.0% (47.2–61.8%).

### Classification prognostic score development and internal validation

The results of the multivariable Cox model used to develop the NEP-Score are reported in Table 2, together with the results of the univariable Cox model including NEP-Score.

**Table 2:** Results of the multivariable Cox model including the selected covariates and used to develop the prognostic score on training set patients and of the univariable model including the prognostic score.

	Hazard ratio	95% CI	*P* Value
Multivariable Cox model used to develop the prognostic score			
Primary tumor surgery			<0.001
Yes vs no	0.39	0.26–0.57	
Age at metastasis (years)			0.030
46–65 vs ≤45	1.31	0.90–1.89	
>65 vs ≤45	1.74	1.15–2.65	
Primary tumor classification site			0.005
Other GEP-NET vs ileum	2.51	1.53–4.13	
Carcinoid lung vs ileum	2.00	1.19–3.34	
Unknown vs ileum	1.52	0.89–2.60	
Pancreatic vs ileum	1.76	1.10–2.82	
Metastasis timing			0.032
Metachronous ≤24 months vs synchronous	1.98	1.15–3.42	
Metachronous >24 months vs synchronous	1.44	0.87–2.36	
Ki-67 (Mib-1)			0.003
Missing vs 0–2	1.72	1.23–2.42	
3–20 vs 0–2	1.12	0.76–1.67	
Functional status			0.073
Yes vs no	1.35	0.97–1.88	
C statistic (95% confidence interval): 0.696 (0.625–0.767)			
Univariable Cox model including prognostic score			
Prognostic score^†^			<0.0001
Intermediate vs favorable risk group	3.08	1.80–5.26	
Poor vs favorable risk group	6.87	4.06–11.64	
C statistic (95% confidence interval): 0.661 (0.592–0.730)			

^†^Favorable risk group: total score ≤70 points, 10-year OS ≥70%. Intermediate risk group: 70 < total score ≤ 198, 30% ≤ 10-year OS < 70%. Intermediate risk group: 70 < total score ≤ 198, 30% ≤ 10-year OS < 70%. Poor risk group: total score ≥199, 10-year OS <30%. The total score was calculated according to Fig. 2.

CI, confidence interval; GEP, gastroenteropancreatic.

**Figure 2:**
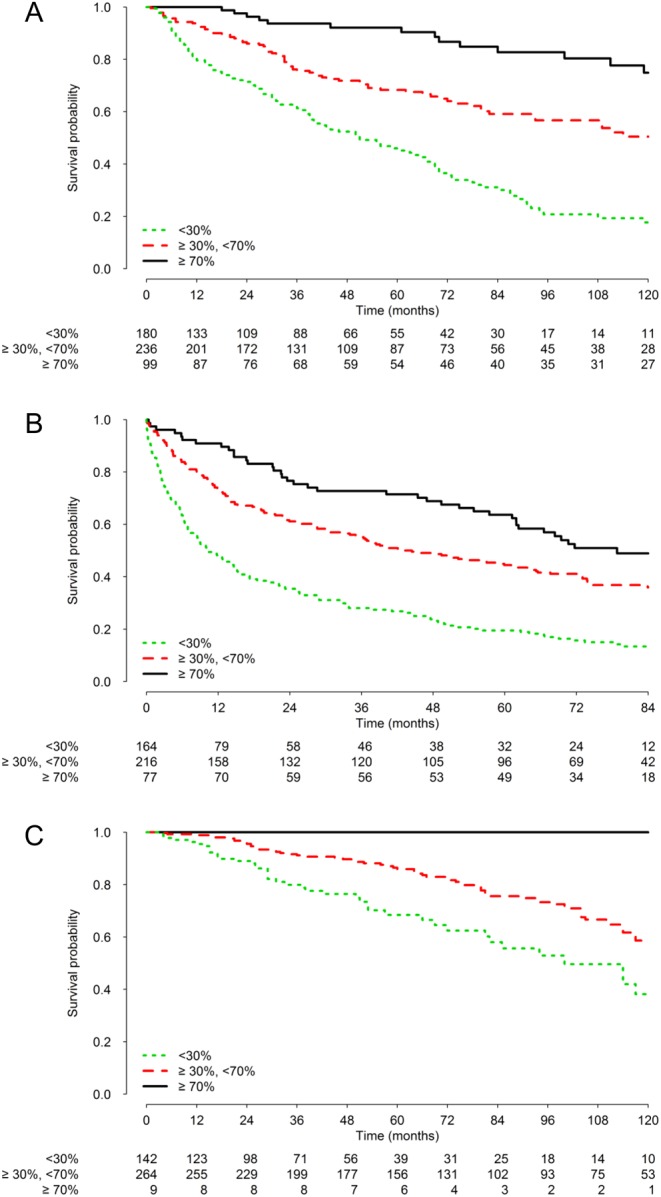
Kaplan–Meier overall survival curves according to the classification prognostic score in training set (panel A) and RARECAREnet and Italian validation sets (panels B and C, respectively). A full colour version of this figure is available at 10.1530/ERC-17-0489.

**Figure 3:**
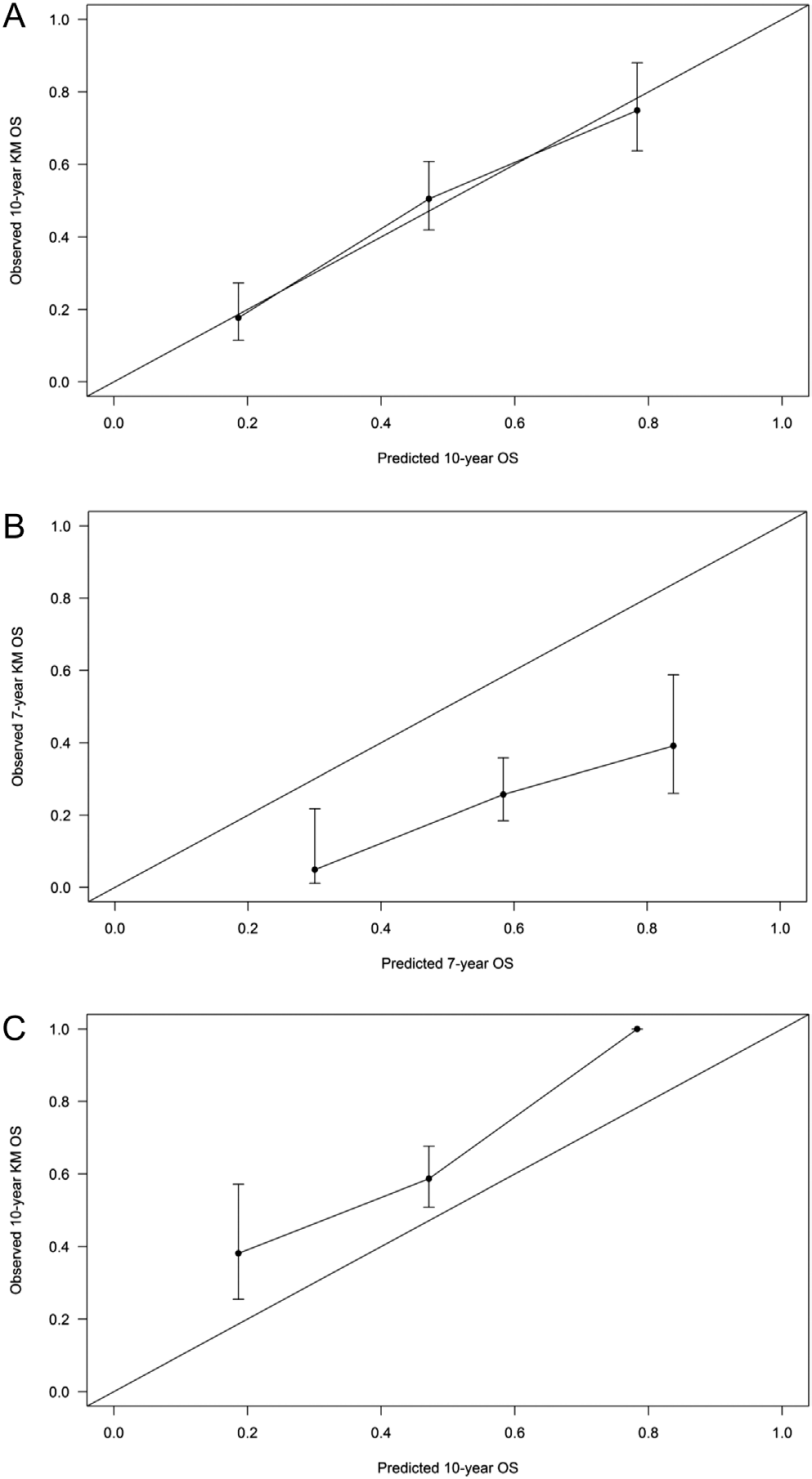
Calibration plot for internal (panel A) and external (RARECAREnet and Italian testing set patients in panel B and C, respectively) validation of the stage IV well-differentiated neuroendocrine prognostic score classification (NEP-Score). The Kaplan–Meier overall survival probability in each prognostic group was plotted (*y* axis) against the corresponding NEP-Score predicted probability (*x* axis). The error bars are the Kaplan–Meier 95% confidence intervals. The solid diagonal line is the reference line, indicating the probability of an ideal classification prognostic score (accordance between predicted and observed probabilities). OS, overall survival.

Table 3 shows the covariate scoring system and how to calculate NEP-Score. This score was able to classify patients into three groups with differential 10-year OS: (I) favorable risk group: total score ≤70 points, 10-year OS ≥70%; (II) intermediate risk group: 70 < total score ≤ 198, 30% ≤ 10-year OS < 70%; (III) poor risk group: total score ≥199, 10-year OS <30%. Table 4 shows the distribution of training set patients and the Kaplan–Meier OS estimates according to the NEP-Score categories, and Fig. 2 (panel A) shows the OS curves. The calibration plot in the training cohort (Fig. 3, panel A) shows very good accordance between the predicted and observed 10-year OS probabilities. The Harrell C statistic for the Cox model including NEP-Score was 0.661 (95% CI: 0.592–0.730) that, being NEP-Score a three-level categorical variable incorporating the prognostic information of multiple variables, was slightly lower than that obtained in the multivariable Cox model from which NEP-Score was generated (0.696 vs 0.661, Table 2).

**Table 3:** Covariate scoring system and NeuroEndocrine Prognostic Score classification (NEP-Score).

Covariate scoring system		
Age at metastasis (years)		Scores
<45		0
46–65		28
>65		58
Primary tumor classification site		Scores
Ileum		0
Unknown		44
Pancreatic		59
Carcinoid lung		72
Other GEP-NET		97
Metastasis timing		Scores
Synchronous		0
Metachronous >24 months		38
Metachronous ≤24 months		72
Ki-67 (Mib-1)		Scores
0–2		0
3–20		12
Missing		57
Functional status		Scores
No		0
Yes		32
Primary tumor surgery		Scores
No		100
Yes		0
**Total score**	**10-year OS**	**Prognostic groups**
Stage IV WD NET patients prognostic score classification (NEP-score)		
*T* ≤ 70	≥70%	Favorable risk group
70 < *T* ≤ 198	30% ≤ OS < 70%	Intermediate risk group
*T* ≥ 199	<30%	Poor risk group

OS, overall survival; total score T, sum of the covariate scores; WD GEP-NET, well differentiated gastroenteropancreatic neuroendocrine tumor.

**Table 4:** Distribution of training and validation set patients and Kaplan–Meier overall survival estimates according to the NEP-Score categories.

	Training set (*n* = 515)	RARECAREnet cohort (*n* = 457)	Italian series cohort (*n* = 415)
Whole series			
5-year, % (95% CI)	64 (60–69)	39 (35–44)	81 (77–86)
10-year, % (95% CI)	43 (37–49)	30 (26–34)^†^	54 (47–62)
NEP-Score*			
Log-rank test *P* value	<0.001	<0.001	0.001
Favorable risk group *n* (%)	99 (19)	77 (17)	9 (2)
5-year, % (95% CI)	92 (86–98)	64 (54–75)	100–
10-year, % (95% CI)	75 (64–88)	39 (26–59)^†^	100–
Intermediate risk group *n* (%)	236 (46)	216 (47)	264 (64)
5-year, % (95% CI)	68 (62–76)	44 (38–52)	86 (81–91)
10-year, % (95% CI)	51 (42–61)	26 (18–36)^†^	59 (51–68)
Poor risk group *n* (%)	180 (35)	164 (36)	142 (34)
5-year, % (95% CI)	45 (38–54)	20 (14–27)	68 (59–79)
10-year, % (95% CI)	18 (12–27)	5 (1–22)^†^	38 (26–57)

*Favorable risk group: total score ≤70 points, 10-year overall survival ≥70%. Intermediate risk group: 70 < total score ≤ 198, 30% ≤ 10-year overall survival < 70%. Intermediate risk group: 70 < total score ≤ 198, 30% ≤ 10-year overall survival < 70%. Poor risk group: total score ≥199, 10-year overall survival <30%. The total score was calculated according to Fig. 2; ^†^7-year OS in RARECAREnet cohort.

CI, confidence interval.

### Classification prognostic score external validation

As compared with the training set, OS was poorer in the RARECAREnet validation set (Fig. 2, panel B); as a consequence, the calibration analysis (Fig. 3, panel B) showed that, when applying NEP-Score to this validation set, in each of the three classification prognostic score categories, the observed OS was slightly overestimated.

As compared with the training set, OS showed an improvement in the Italian multicentric validation set (Fig. 2, panel C); as a consequence, the calibration analysis (Fig. 3, panel C) showed that NEP-Score slightly underestimated the observed OS when applied to this validation set of patients. The Harrell C statistic for the three-levels score was 0.626 (95% CI: 0.571–0.681) and 0.601 (95% CI: 0.505–0.697) in the RARECAREnet and Italian multicentric validation sets, respectively.

#### Complementary analysis

The NEP-Score considered only the prognostic characteristics of patients at diagnosis and did not analyze the impact of the medical treatments received by the patients during the course of the disease.

However, since peptide receptor radionuclide therapy (PRRT) has been shown to prolong OS in midgut NET (interim analysis of the NETTER-1 trial) (Strosberg *et al*. 2017), unlike other approved therapeutic agents that improved progression-free survival only (Yao *et al*. 2016 2017, Faivre *et al*. 2017, Pavel *et al*. 2017, Rinke *et al*. 2017), we speculated that this effect could also be observed in the Italian multicentric validation set enrolling only pNET patients. Moreover, only 7.2% of patients in the training set had received PRRT, vs 25.1% of the multicenter Italian validation set (Table 1).

**Figure 4:**
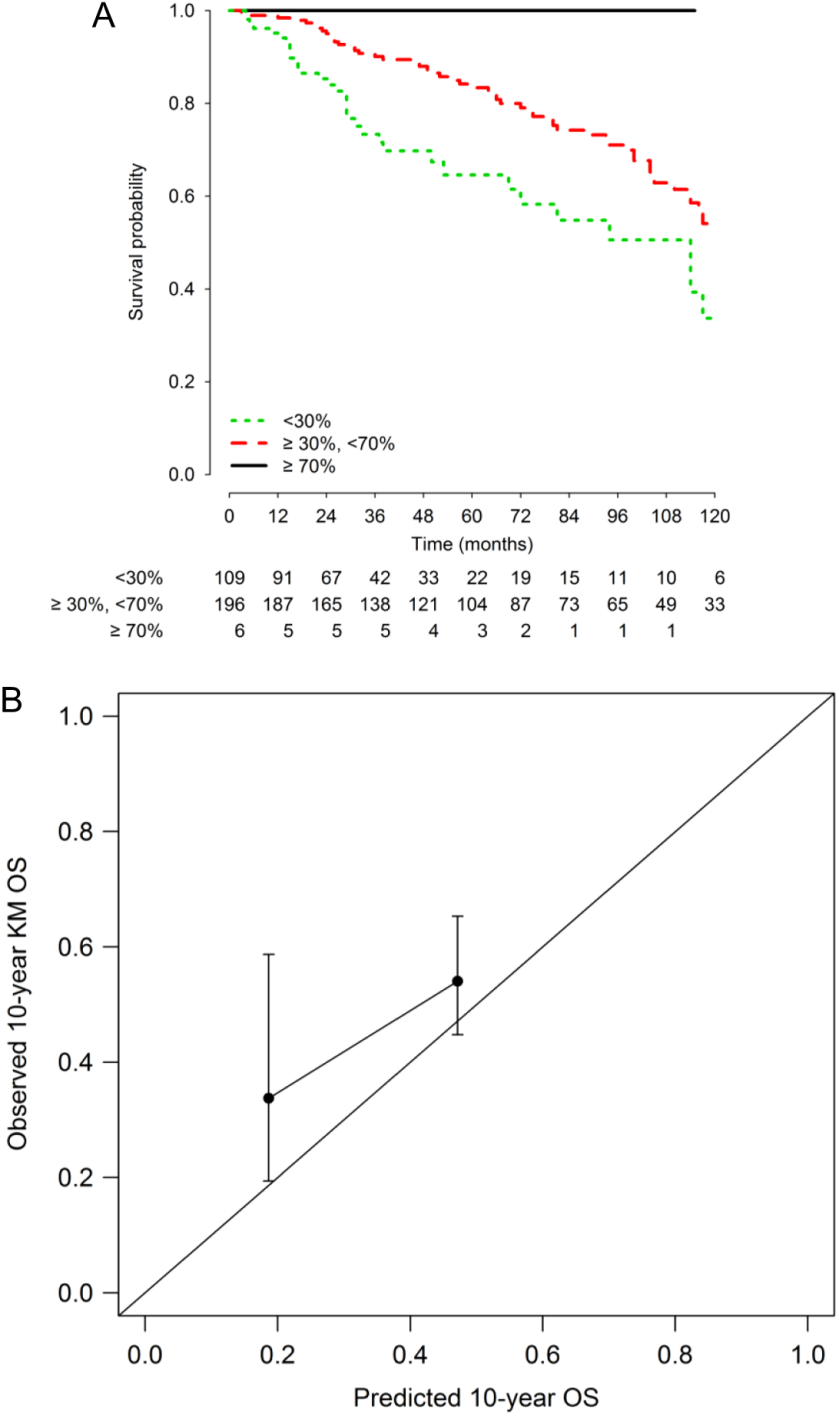
Kaplan–Meier overall survival curves according to the prognostic score classification and calibration plot (panel A and B, respectively) for Italian series cohort patients not treated with peptide receptor radionuclide therapy. A full colour version of this figure is available at 10.1530/ERC-17-0489.

Therefore, as a complementary analysis, we extracted a subgroup of 311 patients not receiving PRRT from the Italian multicentric validation set in order to exclude any potential effect of PRRT on survival. OS curves obtained on patients not receiving PRRT in the Italian validation set are shown in Fig. 4, panel A. 5- and 10-year OS (95% CI) were 78.0% (72.8–83.7%) and 49.2% (41.0–58.9%), respectively. As compared with the calibration plot of the whole Italian validation set, estimated OS is closer to the observed OS (Fig. 4, panel B). No events were observed in the favorable risk group. The Harrell C statistic was 0.626 (95% CI: 0.571–0.681) in this subset.

## Discussion

NETs are an extremely heterogeneous class of neoplasms, and several different tumor- and patient-related factors influence prognosis. Therefore, scores able to define prognosis would be of the highest interest in current clinical research and practice on NETs.

Current treatments for G1–2 metastatic WDNETs include somatostatin analogues (SSAs), chemotherapy, targeted therapies and PRRT, without a precise definition of the best sequence (Kulke *et al*. 2008, Rinke *et al*. 2009, Yao *et al*. 2010, 2011, 2016, 2017, Pavel *et al*. 2011, Strosberg *et al*. 2012, 2017). Treatment selection is therefore based on the evaluation of tumor and patient characteristics – also because of the lack of randomized trials due to the heterogeneity and rarity of disease – in a tailored approach.

Although most WD-lung NETs and patients with WD-GEP-NET are characterized by an indolent disease, a minority of them show a poor outcome and shorter survival with an unpredictable clinical course. However, with standard therapeutic options, median progression-free survival is generally extended by less than six months due to the development of resistance, and benefit is mainly limited to disease control, which eventually results in disease progression.

Noteworthy, we still do not know what biological, pathological or clinical features might be able to characterize this subgroup of ‘poor risk’ patients within the G1–G2 classification and therefore be able to provide a recommendation for the best algorithm for treatment.

To this end, no prognostic tools have been specifically developed to estimate OS probability in specific subgroups of patients with G1–2 NETs. In this large study, based on the analysis of three ‘field-practice’ cohorts collected in different settings, we defined and validated a prognostic score able to classify stage IV WD NET patients into three groups with different long-term prognosis (10-year OS). NEP-Score takes into account some immediately retrievable factors, namely age, primary tumor site, metastasis (synchronous vs metachronous), Ki-67, the presence of functioning status and prior surgical removal of the primary tumor, and therefore, may be easily applicable in clinical practice. According to the factors mentioned earlier, this score stratifies patients into a favorable risk group (OS ≥70%), an intermediate risk group (30–70%) and a poor risk group (<30%). Of note, molecular factors influencing prognosis are not yet validated for NETs and therefore they were not considered for the development of this tool. Moreover, given the limited proportion of patients in the training set with a single metastatic lesion (8.3%) or extra-hepatic disease only (18.6%), we decided not to include them in the multivariable Cox model used to develop the prognostic score. Therefore, we analyzed single vs multiple sites of disease. However, no specific substaging of stage IV exists to help quantify metastatic disease, and therefore, this parameter would be difficult to utilize in a scoring system such as ours. NEP-Score was developed in a monocentric cohort of patients referring to an Excellence Center for the treatment of NETs, thus ensuring a high level of reliability of data homogeneity. Then, NEP-Score was validated in two external cohorts, the former European and the latter Italian multicentric group. The European cohort showed a lower OS than in the training set, which is not surprising. It is well known that OS at the population level is lower than that in clinical series, since population data include information on all hospital settings (general and specialized), as well as on patients not accessing the hospital, and of any age. Our OS were coherent with those reported in a similar population-based study (Yao *et al*. 2008). Thus, such a characteristic is independent of the prognostic variables and was associated with a lower baseline OS which, in turn, affected the calibration results (i.e. NEP-Score slightly overestimated the observed OS). Nevertheless, the calibration plot points were aligned and parallel to the reference line, indicating that the predictions were systematically too high and the covariates had a similar effect in the validation set. On the other hand, patients in the Italian validation cohort showed a higher OS when compared with those of the validation set and the calibration plot points were aligned and parallel to the reference line, thus indicating that the predictions were systematically too low, and even in this case, the covariates had a similar effect in the validation set.

We cannot rule out that this difference can be justified, at least in part, by the inclusion of pNET patients solely in this cohort, and in particular, by the opportunity, for these patients, to access more effective treatments and be included into clinical trials with respect to unknown primary/midgut or lung NET patients (87.3% and 73% of patients including in the training set and RARECARE validation set, respectively). In addition, patients included in the Italian multicenter set were treated from 2000 to 2015, while those in the training set were treated from 1988 to 2012 and those in the RARECAREnet from 2000 to 2007. Therefore, patients in the Italian multicenter set could have received a more refined diagnosis and treatment compared with others. Due to the marked variability and availability of treatments in recent decades and different countries, we did not evaluate the impact of medical treatments received by patients on survival. Moreover, since PRRT recently showed a prolongation of OS in midgut NET patients (Strosberg *et al*. 2017), while other available treatments did not (Rinke *et al*. 2009, Yao *et al*. 2016, 2017, Faivre *et al*. 2017, Pavel *et al*. 2017), we speculated that this effect could also be observed in pNET patients and that this could be another of the causes of overestimated OS in the multicentric Italian validation cohort.

Therefore, in order to partially address this issue, we conducted a subgroup analysis excluding patients who received PRRT. Despite the limited number of patients in this subgroup and the consequent poor precision of calibration analyses, we found a therapeutic factor able to prolong OS, since the calibration plot points are approaching the reference line (Fig. 4). Of note, calibration and discriminative ability are referred to different aspects of model performance and, as in our case, the slight deviance from the perfect calibration in the two validation cohorts (with a good parallelism of the curves with the reference line, indicating that the covariates’ effects were correctly estimated by the NEP-Score model) does not affect the discriminative ability, which was comparable in the training and validation cohorts. Thus, NEP-Score can also be applied to stratify survival probability in different and heterogeneous groups of patients. We must however acknowledge some limitations of our study, including those inherent to any retrospective observational study with a long observation period (e.g., poor reporting of data). Moreover, although very recent studies have paved the way for a deeper investigation of molecular prognostic factors in NETs (Scarpa *et al*. 2017), at the moment of the conduction of the present analysis molecular prognostic factors for this class of neoplasms were not validated yet, and therefore, were not considered for the development of NEP-Score. Patients were recruited over different and prolonged time periods (1988–2012 in the training set), and therefore, we cannot rule out that improvement of care over time may have somehow biased our findings. Future studies could contribute to update NEP-Score in two ways: by simplifying it even more than it already is without lose in discriminative ability or by adding molecular prognostic factors, thus increasing discriminative ability.

Despite these limitations, we were able to develop, by analyzing a large population of patients, an easy and inexpensive scoring system, which might support clinicians in clinical decision-making. We acknowledge that our score is more complex than the commonly used approach of including patients with progressive disease over a specified time period (such as 6, 12 or 36 months). However, our score takes into account several different pieces of information that may allow to identify patients more suitable for clinical trials. Indeed, our score is based on a hard endpoint – OS – and includes several clinical variables associated with time to progression. Moreover, current treatment algorithms for the therapy of NET do not provide recommendations on the sequence of therapy. The challenge is to predict the aggressiveness of individual tumors in order to identify WDNET patients who will benefit from ‘early aggressive’ therapy and to minimize harm from the inadvertent overtreatment of patients with indolent disease. Therefore, we speculate that the stratification of patients according to NEP-Score may be useful in the definition of a tailored therapeutic strategy, e.g., by initiating an early intensive treatment (targeted therapies, chemotherapy or PRRT) in patients with poorer prognosis (i.e., ‘poor risk’ group) or saving more tolerable therapies like SSAs for those with indolent disease, who have a predicted longer survival (‘favorable risk group’) and may require long-lasting therapy. Indeed, a well-established tailored approach should be based on the proper evaluation of the risk of adverse events and the presence of comorbidities vs therapeutic strategy and life expectancy. Since treatment of NETs is often prolonged, it is crucial to avoid those mild-to-moderate adverse events which, when persisting, could lead to worsening of patient’s quality of life.

Clearly, further clinical trials are required to explain the precise strategy and the optimal specific sequence and timing of standard therapeutic options. This clinical classification prognostic score, validated in a large population of patients, may represent a useful tool for the design of prospective clinical trials aimed at assessing the effect of different treatments and their appropriate sequence in different risk groups of WDNET. Moreover, a future challenge will be to integrate biological and epigenetic characteristics of the tumors into well-tested prognostic models.

## Supplementary Material

Supplementary Methods

## Declaration of interest

L G declares relationships with Bayer, Eisai, Otsuka, Helssinn, LeoPharma, Grunenthal, Pierre-Fabre, Indena, Abbvie, CSL Behring, Santhera and Recordati. SP declares relationships with Ipsen, Novartis, Italfarmaco, Pfizer and Advanced Accelerator Application (AAA).

## Funding

This work did not receive any specific grant from any funding agency in the public, commercial, or not-for-profit sector.

## Authors contribution statement

R M and F B performed the statistical analysis. A T, G G, L G, R M and S P designed the research. R M, S P and L G drafted the manuscript. All authors contributed to the revision/editing of the manuscript and approved the final version.
