# Supplementary material for: A classification prognostic score to predict OS in stage IV well-differentiated neuroendocrine tumors
Source: Endocr Relat Cancer. 2018 Mar 20;25(6):607–18. doi: 10.1530/ERC-17-0489 (PMC5920017; doi:10.1530/ERC-17-0489)
Supplement: Supplementary Methods [file erc-25-607-s001.pdf]

Supplementary Statistical Methods

Classification prognostic score development and validation

The NEP-Score was intended to classify patients according to their predicted 10-year OS.

We firstly estimated the training set patient-specific 10-year OS probability from the final Cox model described in the "Statistical Methods" section. From the same model we derived a covariate scoring system: for each covariate category we calculated an integer score proportional to the Cox model coefficient; the covariate score expresses the strength of the relationship between the covariate category and OS, greater values corresponding to lower OS, with zero associated with the reference category taken as the best prognosis group. Then, for each of the training set patients a total score was calculated as the sum of his/her specific covariate scores. The last step to develop NEP-Score was the search for two cut-off points of the total score allowing segregation of patients into three different groups according to their predicted 10-year OS: i) favorable risk group: OS ≥70%; ii) intermediate risk group: OS ≥30% and <70%; iii) poor risk group: OS <30. The two cut-off points 30% and 70% are the rounded values closest to the 1st and 2nd tertiles of a survival probability distribution uniform in the interval (0, 1). In each of the three classes the total score was bounded in an interval by finite limits, as shown in Table 3.

The testing sets patients were scored based on NEP-Score scoring system and classified in each of the three classes.

The NEP-Score performance was evaluated by examining calibration (calibration plot) and discriminative ability (Harrell C index) (Harrell 1996) on the training set (internal validation) and on the testing sets (external calibration).

Calibration refers to the level of agreement between predicted and observed outcome. In a survival analysis context it is typically assessed by reviewing the plot of the mean of the survival probabilities, predicted according to a regression model or other prediction rule at a selected time in different patient groups, versus the corresponding Kaplan-Meier survival probability. For a perfectly calibrated model predicted and observed probabilities would agree falling along a 45-degree line. The points of a calibration plot may be linearly interpolated; a line intercept different than zero indicates predictions systematically too low or too high, and a slope different from 1 reflects a need for shrinkage of the prediction model regression coefficients due to overfitting, and/or a different covariate effect (Steyerberg et al 2010). Our calibration plots represented only three points, being NEP-Score a three-level categorical variable. In each of the three analyzed sets

(training and testing) the observed probabilities (vertical axis of the calibration plot) were the 10-year Kaplan-Meier survival probabilities estimated in the three NEP-Score groups. As regards the predicted probabilities, for the training set they were the 10-year OS probabilities estimated by a univariable Cox model fitted on the training set and including the three-level NEP-Score categorical variable. For each of the testing sets the predicted probabilities were estimated according to the above univariable Cox model fitted on the training set. In each series, all the patients belonging to one of the three NEP-Score categories shared the same predicted probability (horizontal axis of the calibration plot).

The discriminative ability refers to the ability of the regression model or other prediction rule to distinguish subjects with different outcomes. An excellent model gives predicted probabilities close to 100% for patients with the outcome and probabilities close to 0% for patients without the outcome, hence the more it will show a widespread in the distribution of the predicted probabilities the better. For binary outcomes the discriminative ability may be quantified by means of the Area Under the Receiver Operator Curve (ROC-AUC, the higher towards 1 the better). The Harrell C index is an extension of the ROC-AUC suitable for survival data; we estimated the index together with its 95% confidence interval on an individual patient basis, as transformation of the Somers' Dxy rank correlation between the predicted OS probability and the observed censored response variable. Two Harrell C estimates were obtained, one using the predictions from the final (multivariable) Cox model and the other using the predictions from the univariable Cox model including the three-level NEP-Score categorical variable (Table 2).

References

- Harrell FE Jr, Lee KL & Mark DB 1996 Multivariable prognostic models: issues indeveloping models, evaluating assumptions and adequacy, and measuring and reducing errors. *Statistics in medicine* **15** 361-387.
- Steyerberg EW, Vickers AJ, Cook NR, Gerds T, Gonen M, Obuchowski N, Pencina MJ & Kattan MW 2010 Assessing the performance of prediction models: a framework for traditional and novel measures. *Epidemiology* **21** 128-138.
